# First Case of Human Rabies in Chile Caused by an Insectivorous Bat Virus Variant

**DOI:** 10.3201/eid0801.010108

**Published:** 2002-01

**Authors:** Myriam Favi, Carlos A. de Mattos, Verónica Yung, Evelyn Chala, Luis R. López, Cecilia C. de Mattos

**Affiliations:** * Instituto de Salud Pública, Ministerio de Salud Pública, Santiago, Chile; † Centers for Disease Control and Prevention, Atlanta, Georgia, USA; ‡ Hospital Clínico Fusat of Rancagua, Rancagua, Chile

**Keywords:** rabies, Chile, bats, human, Latin America, *Tadarida*

## Abstract

The first human rabies case in Chile since 1972 occurred in March 1996 in a patient without history of known exposure. Antigenic and genetic characterization of the rabies isolate indicated that its reservoir was the insectivorous bat *Tadarida brasiliensis*. This is the first human rabies case caused by an insectivorous bat rabies virus variant reported in Latin America.

In Latin America, rabies in bats was suspected during the 1910s in Brazil and was definitively diagnosed for the first time in Trinidad in 1931 (*1*,*2*). Since then, rabies has been diagnosed in numerous species of nonhematophagous bats throughout this region (*3*). Despite these early discoveries, the important role of nonhematophagous bats in the epidemiology of the disease remained overshadowed by the presence of canine and vampire bat rabies in the region (*4*). During the past decade, with the control of dog rabies in many urban areas and the incorporation of antigenic and molecular typing of viral variants into rabies surveillance programs, an appreciation for the importance of nonhematophagous bats in rabies epidemiology began to emerge in Latin America (*5*–*9*). Rabies virus has been isolated frequently from insectivorous and frugivorous bats in cities across Latin America (*5*,*10*–*12*). This situation also characterizes the current epidemiologic pattern of rabies in Chile, where dog rabies has been controlled. The last human rabies case in Chile caused by a dog bite occurred in 1972 (*5*); since 1985, insectivorous bats have been the main rabies reservoirs identified. As such, these bats are the most important source of infection for the sporadic rabies cases diagnosed in domestic animals every year (*5*). In 1996, after a period of 24 years with no known human rabies deaths, the first human rabies case with an insectivorous bat as the source of infection was reported in Chile (*13*).

## Case Report

**Figure 1:**
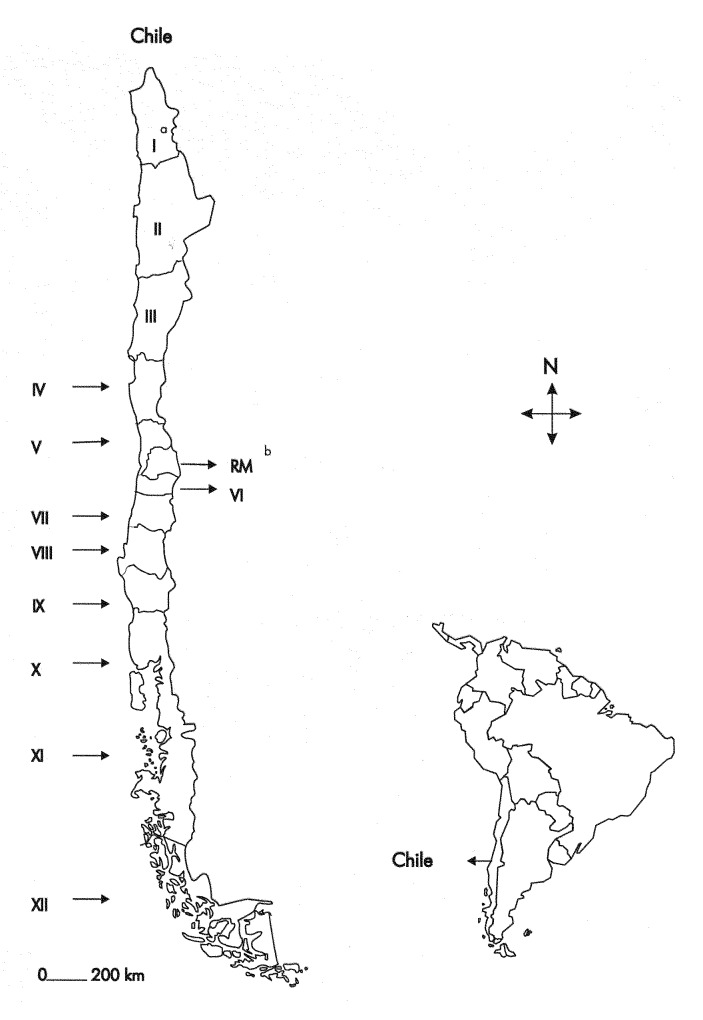
Map of South America showing the geographic position of Chile and map of Chile presenting the geographic distribution of the administrative regions of the country. ^a^Number of the corresponding administrative region. ^b^Metropolitan region.

On February 13, 1996, a 7-year-old boy from Doñihue in Administrative Region VI was admitted to the Hospital Clínico Fusat of Rancagua in the region (Figure 1) with a 2-day history of adynamia and dizziness. On admission, the child was calm, cooperative, and afebrile. Physical examination revealed anisocoria, ptosis of the left upper eyelid, and strabismus. There was no sensory loss, but ambulatory difficulties and abundant sialorrhea were observed. Brain computerized axial tomography (CAT) scan was normal. Polyradiculoneuritis was suspected, and gamma globulin was administered intravenously. The presumptive clinical diagnosis was encephalitis. On February 15, progressive paralysis developed that evolved to respiratory failure; the boy was connected to a mechanical ventilator. The patient could still follow simple orders. On February 18, he lapsed into a coma with severe hypotonia and total loss of reflexes. CAT scan showed diffuse cerebral edema, and the electroencephalogram indicated no electric activity. Intracranial hypertension developed, and the patient was put under hyperventilation and treated with intravenous dexamethasone, mannitol, and acyclovir.

Since a virus was considered the most probable cause, laboratory tests were conducted to determine the presence of the following viruses: herpes, measles, Coxsackie, echo, and polio. All results were negative. Interviews with relatives and the boy’s nanny revealed that bats had been observed in the family’s house. The nanny also reported that she had seen a bat flying away from the child’s toy box. Even though these interviews failed to reveal any direct contact with bats or any history of an animal bite, this epidemiologic information prompted the physicians to suspect rabies. On February 26, 1996, a serum sample and corneal smear were obtained from the patient and sent to the Rabies Laboratory of the Instituto de Salud Pública de Santiago (ISP). A rabies antibody titer of 1:625 was found in the serum specimen by using the indirect fluorescent-antibody (IFA) technique (*14*). The patient had no history of rabies vaccination to account for the presence of antibodies. The corneal smear was negative for rabies antigen by the direct fluorescent-antibody (DFA) assay (*15*). On March 4, a second serum sample, cerebrospinal fluid, and saliva were obtained. The second serum sample was tested simultaneously with the first one by IFA assay, and a titer of 1:15,625 was detected. The cerebrospinal fluid showed a titer of 1:125. These findings confirmed the presumptive clinical diagnosis of rabies. The saliva sample was negative by DFA assay and suckling mouse inoculation (*15*,*16*).

The patient died on March 5, 1996, when artificial respiratory support was disconnected. Postmortem tissue samples of cerebral cortex, hippocampus, cerebellum, and nuchal skin biopsy were sent to the ISP Rabies Laboratory for diagnosis. The cerebellum and skin specimens were positive for rabies virus antigen by DFA assay.

Rabies postexposure prophylaxis with the suckling mouse brain Fuenzalida-Palacios vaccine was administered to the victim’s mother and to 10 health-care providers who had possible contact with the patient’s saliva. The rabies postexposure prophylaxis schedule used was 2 mL of vaccine, subcutaneously, on each of days 1, 2, 3, 4, 5, 6, 21, and 90. Blood samples were taken from vaccinees on day 14 after the initial dose of vaccine; IFA assay showed that adequate immune responses had developed.

The virus was isolated from the patient’s brain tissue by intracerebral inoculation of suckling mice (*16*). To help identify the possible source of infection, the virus was antigenically and genetically characterized. Antigenic characterization of the virus was carried out by using a panel of eight monoclonal antibodies directed against the viral nucleoprotein, provided by the Centers for Disease Control and Prevention. The MAbs were used in an IFA assay as described (*9*,*17*). These analyses identified a rabies antigenic variant associated with *Tadarida brasiliensis* (free-tailed bat) in Chile, which had been designated as antigenic variant 4 (AgV4) (*9*,*17*).

Genetic characterization was done by sequencing a 320-bp portion of the rabies virus nucleoprotein gene from nucleotide position 1,157 to 1,476, as compared with the SADB 19 strain (*18*,*19*). Briefly, genomic viral RNA was extracted from infected tissue by using TRIzol (Invitrogen, San Diego, CA, formerly GIBCO-BRL Inc.) according to the manufacturer’s instructions. Complementary DNA was produced by a reverse transcription polymerase chain reaction with primers 10 g and 304 (*19*) and was sequenced by using the Taq Big Dye Termination Cycle Sequencing Ready Reaction Kit (Applied Biosystems, Foster City, CA), according to the manufacturer’s protocol, on an Applied Biosystems 377 DNA automated sequencer (Applied Biosystems).

This human rabies virus isolate was compared with viruses obtained from domestic animals and insectivorous bats in urban centers in Chile from 1977 to 1998 (*18*). PileUp and Pretty programs of the Wisconsin Package, Version 10 (Genetic Computer Group, 2000, Madison, WI), were used to produce sequence alignments and comparative nucleotide analyses. The programs DNADIST (Kimura-two parameter), NEIGHBOR (Neighbor-joining method), and DNAPARS (parsimony method) from the PHYLIP package, Version 3.5 (*20*), were used in the phylogenetic studies. The bootstrap method, as implemented by the SEQBOOT program from PHYLIP, was followed by the use of DNADIST and NEIGHBOR for the distance matrix analyses. SEQBOOT was also used before employing DNAPARS for the parsimony studies. Graphic representation of the trees was constructed with the TREEVIEW program (*21*).

**Figure 2:**
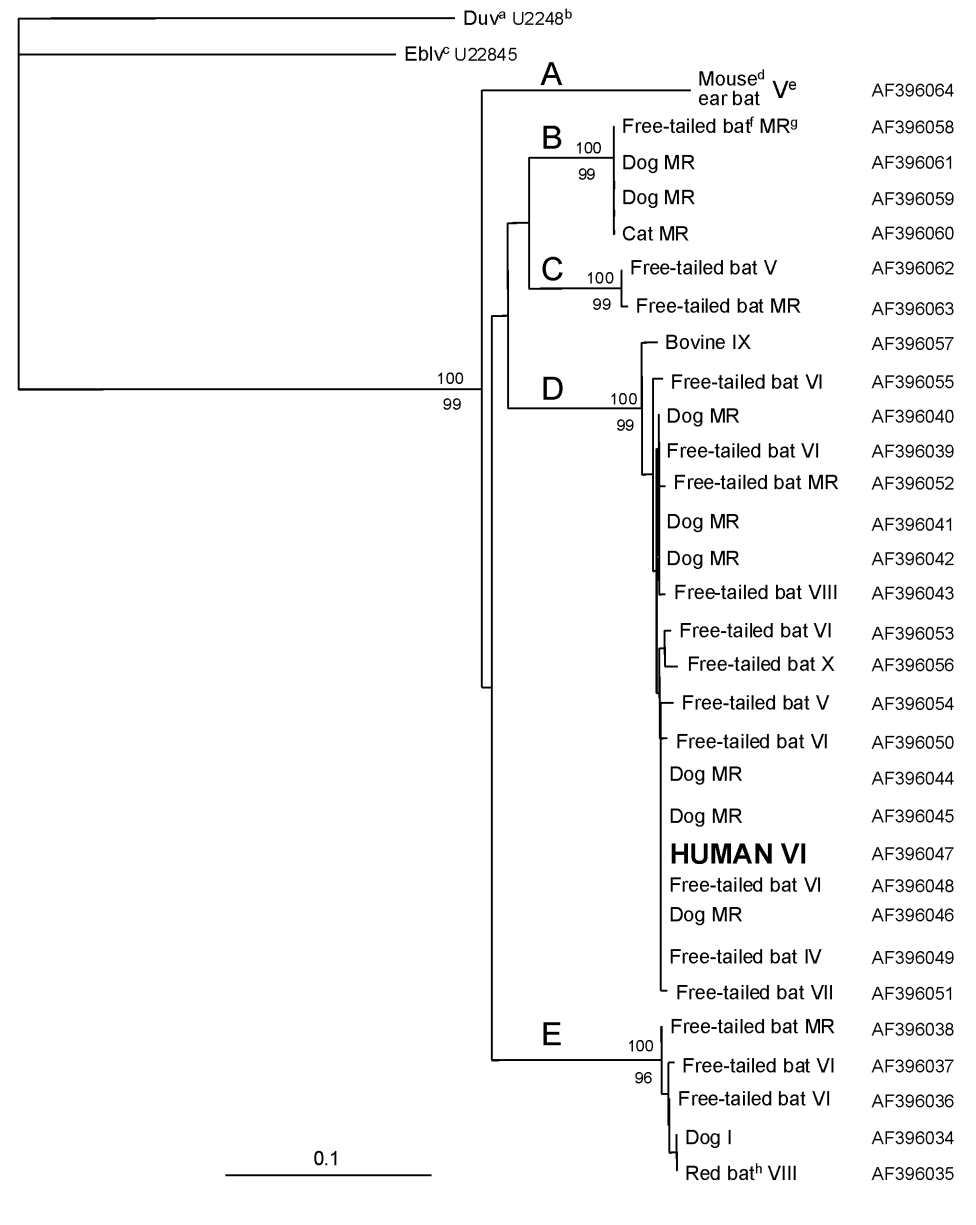
Neighbor-joining tree comparing the human rabies isolate with representatives of the rabies genetic variants obtained from insectivorous bats and domestic animals in Chile (*18*). Bootstrap values obtained from 100 resamplings of the data by using distance matrix (top) and parsimony methods (bottom) are shown at nodes corresponding to the lineages representing the rabies virus variants (A, B, C, D, and E) currently circulating in Chile. Only bootstrap values >50% are shown at the branching points. The bar at the left corner indicates 0.1 nucleotide substitutions per site. ^a^Duvenhage virus, ^b^GenBank accession number, ^c^European bat Lyssavirus, ^d^*Myotis chiloensis*, ^e^Roman numerals indicate the administrative region where the sample was obtained, ^f^*Tadarida brasiliensis*, ^g^Metropolitan region, ^h^*Lasiurus borealis*.

Although five genetic variants of rabies virus are found in Chile (*18*) (Figure 2, groups A to E), a reservoir has been identified for only two: *T. brasiliensis* (Figure 2, group D) and *Lasiurus* sp. (Figure 2, group E). Phylogenetic analyses of the Chilean human isolate demonstrated that it segregated in group D. This group represents the genetic variant of rabies virus most frequently isolated throughout the country, formed by viruses from the Metropolitan Region and Regions IV, V, VI, VII, VIII, IX, and X (Figure 1). The high bootstrap value that supports the inclusion of this virus in group D and the very close genetic relationship it has with the other members of this group (average genetic distance 0.5%) clearly show that *T. brasiliensis* is the likely reservoir of the rabies virus isolated in this case.

## Conclusions

The absence of a history of an animal bite, the clinical presentation of the disease without the classic signs of hydrophobia or aerophobia, and the absence of any human rabies cases for a period of 24 years in Chile were the primary reasons that rabies was not first suspected and a definitive diagnosis was delayed in this case. Retrospective studies of human rabies epidemiology have demonstrated that it is not uncommon to observe rabies cases in which there is no history of a bite, mainly in situations involving insectivorous bat rabies variants. For example, of the 17 human rabies cases associated with insectivorous bats reported in the United States from 1980 to 1996, only one had clear documentation of a bite (*22*). Without proper education, patients may not be aware of the risks from a bat bite. Moreover, the wound may not be appreciated as a concern because of the limited injury inflicted by the bat's small teeth (*23*). Finally, there may not be an opportunity to obtain a history from a pediatric patient or to discern an exposure that occurs during sleep or other circumstances (*24*).

In cases in which a patient shows clinical signs of central nervous system involvement of unknown or suspected viral origin, health-care providers should be aware of the importance of conducting a thorough medical history to appropriately assess the possibility of rabies. With the important changes in the epidemiologic patterns of rabies in Latin America, this disease should be included in the differential diagnosis of neurologic diseases characterized by acute encephalitis and progressive paralysis, even when no previous history of an animal bite exists and even in regions where canine rabies has been eradicated.
